# Remimazolam Requires Less Vasopressor Support during Induction and Maintenance of General Anesthesia in Patients with Severe Aortic Stenosis Undergoing Transcatheter Aortic Valve Replacement: A Retrospective Analysis from a Single Center

**DOI:** 10.1155/2022/6386606

**Published:** 2022-10-22

**Authors:** Hirotsugu Miyoshi, Tomoyuki Watanabe, Kenshiro Kido, Satoshi Kamiya, Sachiko Otsuki, Soshi Narasaki, Yukari Toyota, Takashi Kondo, Yousuke T. Horikawa, Noboru Saeki, Yasuo M. Tsutsumi

**Affiliations:** ^1^Department of Anesthesiology and Critical Care, Hiroshima University, Hiroshima, Japan; ^2^Department of Pediatrics, Sharp Rees-Stealy Medical Group, San Diego, California, USA

## Abstract

**Introduction:**

We compared the hemodynamics during general anesthesia with remimazolam and conventional anesthetics in patients with severe aortic stenosis (AS).

**Methods:**

This was a retrospective single-center analysis. We reviewed the records of 42 patients who underwent transcatheter aortic valve implantation with a transfemoral artery approach under general anesthesia from January to December 2020. Patients were divided into three groups based on the general anesthetic used for (induction/maintenance) remimazolam/remimazolam (Group R/R), propofol/sevoflurane (Group P/S), and midazolam/propofol (Group M/P). Vasopressor use (ephedrine, phenylephrine, and noradrenaline) was compared among the groups.

**Results:**

The number of patients in each group was 15 (Group R/R), 13 (Group P/S), and 14 (Group M/P), with no significant difference in background characteristics and intraoperative vital signs. For anesthesia induction, doses of ephedrine and phenylephrine used were significantly lower in Group R/R (ephedrine [mg]: Group R/R 2 [0–4] vs. Group P/S 8 [8–12], *P* < 0.001, Group R/R vs. Group M/P 5 [0–15], *P* = 0.39; phenylephrine (mg): Group R/R 0 [0–0.08] vs. Group P/S 0.15 [0.10–0.20], *P* = 0.03, Group M/P 0.21 [0.04–0.40], *P* = 0.08). For anesthesia maintenance, the noradrenaline dose used was low in the Group R/R (noradrenaline [*μ*g/kg/min]: Group R/R 0.019 [0.015–0.039], Group P/S 0.042 [0.035–0.045], *P* = 0.02, Group M/P 0.048 [0.040–0.059], *P* < 0.01).

**Conclusion:**

In patients with severe AS, induction and maintenance of anesthesia with remimazolam resulted in less overall vasopressor use than conventional general anesthetics.

## 1. Introduction

Remimazolam is an ultrashort-acting sedative/anesthetic with a high affinity for the benzodiazepine binding site of the *γ*-aminobutyric acid (GABA) receptor [1]. Remimazolam is used as a novel intravenous anesthetic for the induction and maintenance of general anesthesia [2]. Remimazolam incorporates an ester linkage in addition to the characteristics of benzodiazepines. Therefore, this anesthetic has a safety profile that is similar to that of midazolam [3]. In addition, remimazolam has on-and-off properties with rapid sedative effects, similar to that of propofol and can maintain a sedative effect with continuous administration [3]. Previous reports suggest that remimazolam maintains stable hemodynamics in addition to the rapid onset and disappearance of the anesthetic and sedative effects; however, its effects during critical cardiac surgery have yet to be described [4–6].

Transcatheter aortic valve implantation (TAVI) is widely used as a minimally invasive treatment for aortic valve stenosis (AS) [7]. Advances in and the widespread use of TAVI have led to the surgical treatment of patients with severe AS patients who were previously untreated [8]. Patients undergoing TAVI have the most severe AS, and in such cases, anesthesia management is challenging. There is no recommended anesthetic for anesthesia management in TAVI, and various anesthetics are administered based on the experience of individual anesthesiologists. There has been one report of remimazolam use in patients with severe AS, but there have been no reports comparing remimazolam to other traditional anesthetics and its ability to maintain stable hemodynamics in patients with severe AS [9].

In our hospital, all TAVI cases are managed under general anesthesia using a standard protocol. Midazolam or propofol is used for anesthesia induction, and propofol or sevoflurane is used for anesthesia maintenance. We hypothesized that remimazolam would require less vasopressor support than the traditional anesthetics. In this study, we compared remimazolam with existing anesthetics in terms of hemodynamics and vasopressor usage during induction and maintenance in patients undergoing TAVI.

## 2. Materials and Methods

This was a retrospective single-center analysis. The study was approved by the appropriate institutional review board (IRB) at Hiroshima University Hospital (Approval E-1463), and the requirement for written informed consent was waived by the IRB. Although a retrospective analysis, bias was limited by following standard anesthesia guidelines. Exclusion criteria were limited to prevent any further bias to the data. Study size was determined by looking at all cases of TAVI during a select period.

### 2.1. Patient Selection

**Figure 1:**
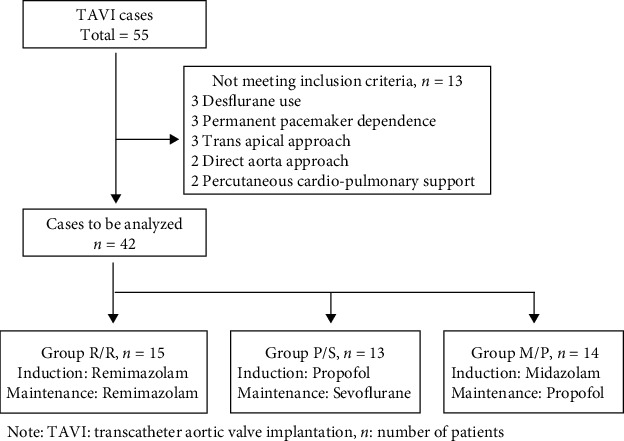
Patient selection criteria. TAVI: transcatheter aortic valve implantation; n: number of patients.

We retrospectively investigated the electronic medical records of patients with severe AS who received general anesthesia for TAVI from January to December 2020 from the Hiroshima University Hospital. We examined cases of TAVI performed using the transfemoral artery approach. We excluded patients who underwent procedures other than the transfemoral artery approach, those who used percutaneous cardiopulmonary support (PCPS), those in whom the heart rate depended on a permanent pacemaker, and those who used desflurane (Figure 1). The subjects were divided into three groups based on the anesthetic used for anesthesia induction and maintenance. Patient hemodynamics and the vasopressor usage were monitored. The three groups were divided as follows: remimazolam was used for anesthesia induction and maintenance (Group R/R), propofol was used for anesthesia induction, sevoflurane was used for anesthesia maintenance (Group P/S), midazolam was used for anesthesia induction, and propofol was used for anesthesia maintenance (Group M/P).

### 2.2. Anesthesia Management Protocol

The standard management protocol for general anesthesia for patients undergoing TAVI at our hospital is shown below. A 22G catheter was inserted into the radial artery prior to anesthesia induction for continuous arterial pressure measurements. The tracheal tube, transesophageal echocardiography, and pulmonary artery (PA) catheter were then placed after the patient lost consciousness. Electrocardiographic findings, direct arterial pressure, saturation of percutaneous oxygen (SpO_2_), Entropy™ (GE Healthcare, Helsinki, Finland), pulmonary arterial pressure, mixed venous oxygen saturation (SvO_2_), and cardiac index (CI) were measured as standard vital signs. Anesthesia was induced with remimazolam, midazolam, or propofol in combination with remifentanil and fentanyl and was maintained with remimazolam or sevoflurane and propofol in combination with remifentanil. Ephedrine, phenylephrine, or noradrenaline was used when the systolic blood pressure dropped below 90 mm Hg or the mean blood pressure was below 60 mm Hg during surgery. The choice of anesthetics used for general anesthesia was determined by the anesthesiologist in charge. Remimazolam was administered at a dose of 12 mg/kg/h, with a maximum of 0.25 mg/kg used for anesthesia induction. Midazolam was administered at 3 mg and propofol was administered at approximately 1 mg/kg, as a bolus intravenous administration, for anesthesia induction. After confirming loss of consciousness, the muscle relaxant rocuronium was administered. Muscle relaxants were reversed using sugammadex in all cases. Remimazolam was reversed by flumazenil (0.2 mg, initial dose, maximum dose 0.5 mg; 0.1 mg increments) when the anesthesiologist determined that the awakening from anesthesia was delayed.

### 2.3. Patient Demographics and Hemodynamics

The patients' demographics include age, sex, height, body weight, body mass index (BMI), and comorbidities such as hypertension, diabetes mellitus, hyperlipidemia, Logistic Euro Score, Society of Thoracic Surgeons (STS) score, Clinical Frailty Scale score, AS severity, aortic valve area, mean pressure gradient (PG), maximum PG, peak velocity, aortic regurgitation graded, and left ventricular ejection fraction (LVEF). We also investigated the dose of anesthetic and analgesic used (remifentanil, fentanyl, remimazolam, midazolam, propofol, and sevoflurane) and the dose of vasopressor and vasodilator used (ephedrine, phenylephrine, noradrenaline, and carperitide). Hemodynamics was continuously monitored following induction until extubation.

### 2.4. Statistical Analysis

We compared the above survey factors among the three groups utilizing the Kruskal-Wallis test to determine any significant differences. We then performed a Mann–Whitney *U* test on any statistically significant groups with a Bonferroni correction to identify any individual significant groupings. Fisher's exact probability test was used to identify nonrandom associations, with the significance set at *P* < 0.05. Data are shown as the median [interquartile range]. PRISM 9.0 software (GraphPad Software, San Diego, CA, USA) was used for all statistical analysis.

## 3. Results

During the study period, 55 patients underwent TAVI, of which 13 patients were excluded. We analyzed the records of 42 patients, of which 15 were in Group R/R, 13 in Group P/S, and 14 in Group M/P. (Figure 1).

Patient demographics revealed no significant difference among the three groups including patient comorbidities and AS severity (Table 1). Furthermore, no significant difference was noted in total operating room time (Data not shown).

The frequency of use of vasopressor agents at the time of anesthesia induction was significantly lower in Group R/R compared to Group P/S (Table 2). In addition, the doses of ephedrine and phenylephrine were significantly higher in Group P/S than in Group R/R although no significant difference was seen compared to Group M/P (ephedrine [mg]: Group R/R 2 [0–4] vs. Group P/S 8 [8–12], *P* < 0.001; Group R/R vs. Group M/P 5 [0–15], P = 0.39; phenylephrine (mg): Group R/R 0 [0–0.08] vs. Group P/S 0.15 [0.10–0.20], *P* = 0.03; Group M/P 0.21 [0.04–0.40], *P* = 0.08). During maintenance anesthesia, the dose of noradrenaline used in Group R/R was lower than that in both other groups (noradrenaline [*μ*g/kg/min]: Group R/R 0.019 [0.015–0.039] vs. Group P/S 0.042 [0.035–0.045], *P* = 0.02; Group R/R vs. Group M/P 0.048 [0.040–0.059], *P* < 0.01).

Table 3 shows the anesthesia event and the time required. During the induction of anesthesia, the time from the start of anesthesia administration to the entropy of less than 60 was significantly shorter in Group P/S than in Group R/R. Furthermore, at the end of anesthesia, the time from stopping the anesthetics to extubation was significantly faster in Group P/S than in Group M/P. (Table 3).

**Figure 2:**
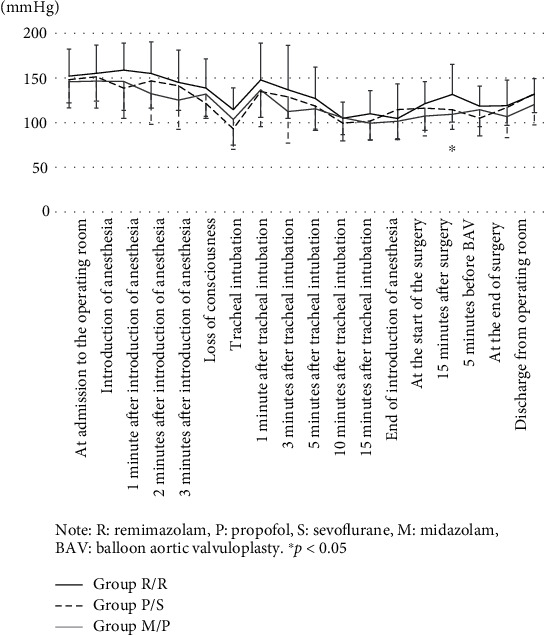
Change in systolic arterial blood pressure over time (mean ± standard deviation). Note: R: remimazolam; P: propofol; S: sevoflurane; M: midazolam; BAV: balloon aortic valvuloplasty. ^∗^*P* < 0.05.

Figure 2 shows the change in systolic blood pressure. There were no significant differences among the three groups at any study points. (Figure 2).

**Figure 3:**
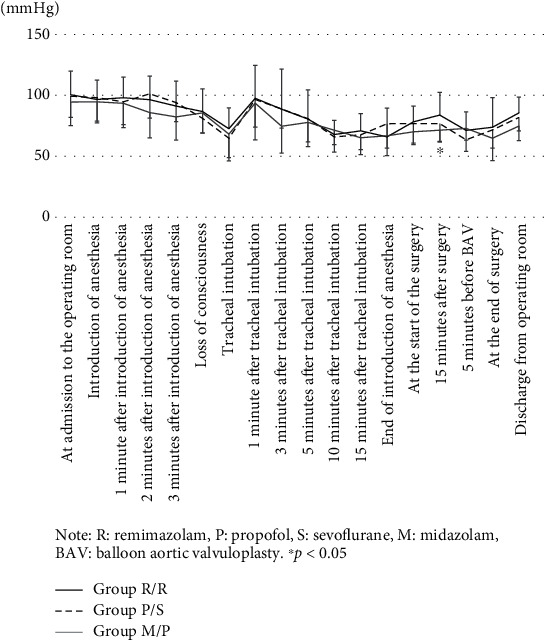
Changes in mean arterial blood pressure over time (mean ± standard deviation). Note: R: remimazolam; P: propofol; S: sevoflurane; M: midazolam; BAV: balloon aortic valvuloplasty. ^∗^*P* < 0.05.

Figure 3 shows the transition of the average blood pressure. The mean blood pressure at the time15 minutes after the start of surgery was significantly higher in Group R/R than in Group M/P. (Figure 3).

**Figure 4:**
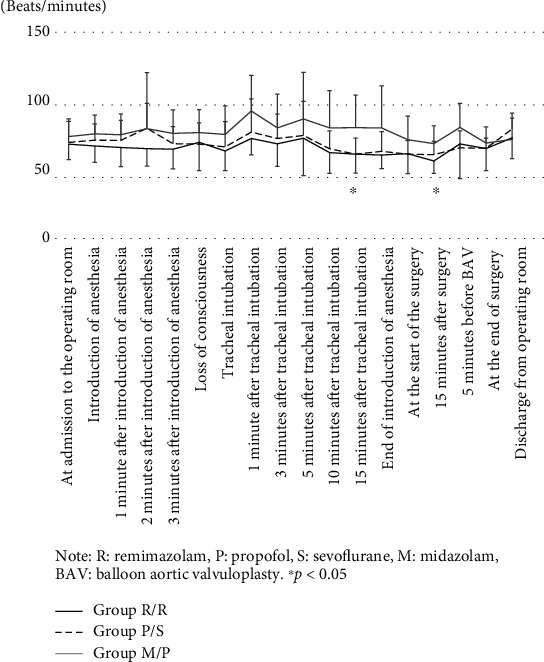
Change in heart rate over time (mean ± standard deviation). Note: R: remimazolam; P: propofol; S: sevoflurane; M: midazolam; BAV: balloon aortic valvuloplasty. ^∗^*P* < 0.05.

Figure 4 shows the transition of heart rate. The heart rate values at the time 15 minutes after tracheal intubation and 15 minutes after surgery were significantly higher in the Group M/P than in the Group R/R. (Figure 4).

**Figure 5:**
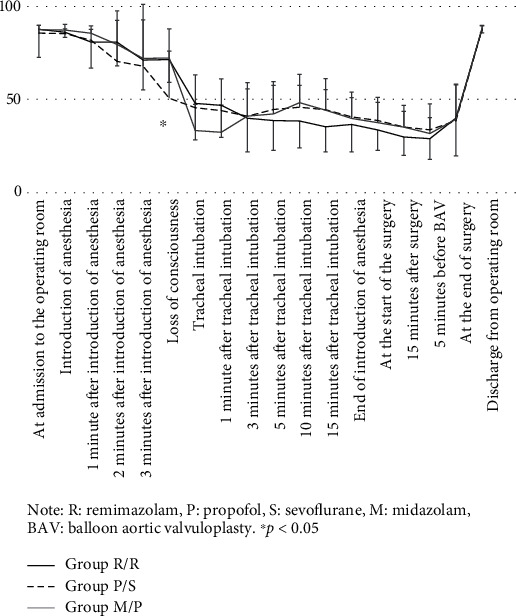
Change in State Entropy over time (mean ± standard deviation). Note: R: remimazolam; P: propofol; S: sevoflurane; M: midazolam; BAV: balloon aortic valvuloplasty. ^∗^*P* < 0.05.

Figure 5 shows the transition of Entropy (State Entropy). The State Entropy value at the time of loss of consciousness was significantly lower in the Group P/S than in the Group R/R. (Figure 5).

Table 4 shows changes in mean PA pressure, SvO2, and CI during surgery. There was no difference in each parameter among the three groups. (Table 4).

## 4. Discussion

We compared the hemodynamics during induction and maintenance of anesthesia between remimazolam and conventional anesthetics in patients with severe AS who underwent TAVI. Remimazolam required overall less vasopressor support of ephedrine and phenylephrine during anesthesia induction. In addition, remimazolam required lesser noradrenaline during maintaining anesthesia. Taken together, remimazolam provided fewer hemodynamic changes than conventional general anesthetics.

Intraoperative hypotension occurs due to various factors, and in recent years, intraoperative hypotension has been reported to affect the postoperative prognosis [10, 11]. Many anesthetics cause vasodilation and cardiac depression due to sympathetic nerve suppression, resulting in severe hypotension after administration, especially in patients with impaired cardiac function and the elderly [12]. Anesthesiologists should understand the characteristics of anesthetics to predict and prevent hypotension. For anesthetic management of patients with severe AS, it is essential to treat hypotension promptly and avoid excessive increases in heart rate. [13–15] Patients with AS often have coronary artery stenosis and myocardial thickening; therefore, low blood pressure can easily result in inadequate blood supply to the myocardium. [13] Hypotension can lead to a reduction in coronary blood flow resulting in further reductions in left ventricular function and cardiac output. In our study, blood pressure was maintained by vasopressor use, and as a result, there was no significant difference in the systolic and mean blood pressures among the three groups. However, 15 minutes after the start of surgery, the blood pressure was significantly higher in Group R/R. In contrast, the heart rate during surgery was higher in Group M/P using propofol to maintain anesthesia than in the remimazolam group. This may be due to the increased use of noradrenaline in Group M/P when using propofol. During anesthesia induction, the doses of ephedrine and phenylephrine were higher in Group P/S. This is consistent with reports that propofol tends to lower blood pressure due to excessive vasodilation, especially in AS patients [12, 16]. However, the Group R/R required less noradrenaline to maintain anesthesia. Furthermore, in the Group R/R, the dose of vasopressor used at the time of induction of anesthesia was minimal, and the proportion of cases requiring vasopressor administration was also small.

At the time of anesthesia induction, we administered remimazolam at 12 mg/kg/h, and the upper limit was set to 0.25 mg/kg to avoid overdose. In our study, it took approximately 130 seconds in the remimazolam group from the start of medication to loss of consciousness, which was slightly slower than previous reports, in which it took about 90 seconds from the start of medication to loss of consciousness [4, 5]. Various factors affect the time required for the onset of drug efficacy. In our study, lower total doses of remimazolam than previously reported may cause delayed efficacy onset. In addition, there may be technical influences such as the method of confirming the loss of consciousness, the infusion circuit, and the difference in infusion rate. It is also possible that, like some muscle relaxants, low cardiac function and differences in the vasopressor drugs used may interact with remimazolam [17, 18]. In patients with severe AS, the onset of the effect of remimazolam may be delayed, and to avoid overdose, it is safest to set an upper limit on the administration. However, the time from administration of the anesthetic to loss of consciousness was similar between Group R/R and Group M/P, consistent with a previous report [19]. This is due to the pharmacologically identical mechanism of action of remimazolam and midazolam [20]. In earlier reports, the onset time was shorter at 12 mg/kg/h than at 6 mg/kg/h; higher doses of remimazolam may result in faster sedation [4, 5]. Remimazolam may be expected to play an active role in rapid sequence induction by adjusting the initial amount.

Interestingly, we found that the value of entropy did not correspond directly to the loss of consciousness in Group R/R with almost a 1 min delay. Group P/S had a decrease in entropy at nearly the same time as the loss of consciousness. Previously, bispectral index prediction of sedation was more difficult with midazolam than propofol [21, 22]. As a result, it is essential to realize that remimazolam or midazolam may have an entropy value that may lag behind loss of consciousness.

Regarding awakening from anesthesia, in our study, there was no difference in the time between discontinuation of medication and extubation between the remimazolam and propofol groups. Propofol metabolism and clearance are rapid, but the metabolism is affected by age and cardiac output, which may result in a slightly delayed arousal in patients with severe AS [23, 24]. However, remimazolam metabolism and clearance are similar to propofol, and its blood concentration decreases rapidly after discontinuation of administration. Remimazolam is metabolized and inactivated by tissue esterases. Since tissue esterase does not depend on the underlying disease, remimazolam has high clearance even in patients with multiple comorbidities [25, 26]. Therefore, remimazolam is useful in anesthesia management for patients with severe AS by decreased cardiac function. Furthermore, flumazenil, a remimazolam antagonist, was administered to 9 of 15 patients in our study [20]. The state entropy level when flumazenil was administered was low at 30, but awakening occurred in approximately 150 seconds. No patient was sedated again after surgery. These results indicate that flumazenil can rapidly antagonize remimazolam even under deep anesthesia.

There are a few limitations to our study. Our study was a retrospective analysis, and much of the anesthesia management was left to the discretion of the individual anesthesiologist [27]. The adjustment of anesthetics, especially for awakening from anesthesia, is greatly influenced by individual preferences among anesthesiologists. To better understand the effects of remimazolam, prospective studies are needed. Furthermore, our sample size is relatively small, and more significant patient populations must identify whether these effects are genuinely significant.

In conclusion, we compared the hemodynamics of patients with severe AS undergoing TAVI with remimazolam and existing anesthetics. During induction and maintenance, anesthesia Group R/R required less vasopressor support than other traditional anesthetics. Remimazolam can maintain stable hemodynamics during anesthesia management of patients with severe AS, with fewer vasopressors.

## Figures and Tables

**Table 1:** Patients' background data. There was no difference between the three groups in terms of patient background factors. There were no differences noted regarding the severity of AS which was determined by mean and max pressure gradients and peak velocity over the valve. Values are shown as median (interquartile range) or number (%). A *P* value < 0.05 was considered to indicate statistical significance. BMI: body mass index; STS: Society of Thoracic Surgeons; TAVI: transcatheter aortic valve implantation; PG: pressure gradient; LVEF: left ventricular ejection fraction.

	Group R/R	Group P/S	Group M/P	*P* value
	*n* = 15	*n* = 13	*n* = 14	
Age (years)	83 [80.5-85.5]	84 [82-86]	82 [80-83]	0.63
Sex male/female (*n*)	3/12	4/9	4/10	0.78
Height (cm)	150 [145-150.5]	149 [144-156]	150 [148-162]	0.56
Weight (kg)	52.8 [44.5-60]	51.7 [44-57]	51.7 [43-64.5]	0.92
BMI (kg/m^2^)	22.0 [21.2-27.2]	22.0 [20.2-25.8]	22.6 [20.0-26.9]	0.59
Logistic EuroScore (%)	11.0 [8.7-14.4]	10.0 [7.6-14.8]	10.7 [9.9-13.2]	0.62
STS score (%)	5.0 [3.7-5.4]	5.0 [3.7-6.8]	5.0 [4.0-7.8]	0.31
Clinical Frailty Scale	4 [4-4.5]	4 [3-4]	4 [3.8-4]	0.70
Indwelling TAVI valve type (SAPIEN3/Evolut-R)	12/3	10/3	12/2	
Aortic valve area (cm^2^)	0.76 [0.50-0.81]	0.70 [0.60-0.80]	0.65 [0.59-0.83]	0.33
Mean PG (mg/dL)	54.8 [41.6-71.0]	50.1 [40.0-69.0]	55.9 [46.0-60.5]	0.85
Max PG (mg/dL)	74.9 [60.5-94.4]	81.4 [70.0-115.0]	82.5 [59.5-89.3]	0.74
Peak velocity (m/s)	4.5 [4.2-4.9]	4.5 [4.2-5.4]	4.6 [4.1-4.9]	0.73
LVEF (%)	65 [61-68]	62 [59-66]	62 [59-64]	0.21
Aortic regurgitation graded more than mild *n* (%)	7 (47)	7 (54)	9 (64)	0.63
Hypertension *n* (%)	11 (73)	12 (92)	10 (71)	0.34
Diabetes mellitus *n* (%)	3 (20)	4 (31)	3 (21)	0.77
Hyperlipidemia *n* (%)	7 (47)	7 (54)	5 (35)	0.63
Pacemaker implanted *n* (%)	0 (0)	1 (8)	3 (21)	0.13

**Table 2:** Anesthetics, vasopressor agents, and vasodilators are used for anesthesia induction and maintenance. Values are shown as median (interquartile range) or number (%). A *P* value < 0.05 was considered to indicate statistical significance. ^∗^*P* value < 0.05 compared to Group R/R; ^∗∗^*P* value < 0.01 compared to Group R/R.

	Group R/R	Group P/S	Group M/P	*P* value
	*n* = 15	*n* = 13	*n* = 14	
Induction of anesthesia				
Fentanyl (*μ*g/kg)	1.7 [0-1.8]	1.5 [0-1.8]	1.5 [0-2.1]	0.87
Remifentanil (*μ*g/kg/min)	0.24 [0.20-0.26]	0.30 [0.24-0.36]	0.33 [0.29-0.36]	0.12
Remimazolam (mg/kg)	0.18 [0.16-0.22]	—	—	—
Propofol (mg/kg)	—	1.06 [0.99-1.14]	—	—
Midazolam (mg/kg)	—	—	0.05 [0.04-0.06]	—
Use of vasopressor (with/without use)	9/6	13/0	12/2	0.02
Ephedrine (mg)	2 [0-4]	8 [8-12]	5 [0-15]	<0.001
Phenylephrine (mg)	0 [0-0.08]	0.15 [0.10-0.20]	0.21 [0.04-0.40]	0.005

Maintenance of anesthesia				
Remifentanil (*μ*g/kg/min)	0.22 [0.19-0.32]	0.15 [0.14-0.19]	0.20 [0.19-0.23]	0.06
Remimazolam (mg/kg/hr)	0.48 [0.30-0.55]	—	—	—
Sevoflurane (%)	—	1.0 [1.0-1.5]	—	—
Propofol (TCI, *μ*g/mL)	—	—	1.0 [1.0-1.12]	—
Noradrenaline (*μ*g/kg/min)	0.019 [0.015-0.039]	0.042 [0.035-0.045]	0.048 [0.040-0.059]	0.002
Carperitide (with/without use)	3/12	4/9	7/7	0.22
Carperitide (*μ*g/kg/h)	1.04 [0.56-1.37]	0.90 [0.75-1.04]	0.94 [0.84-1.04]	0.96

Awakening from anesthesia				
Flumazenil (with/without use)	9/6	—	—	
Flumazenil (mg)	0.5 [0.2-0.5]	—	—	

**Table 3:** Anesthetic events and the time required. Values are shown as median (interquartile range). A *P* value < 0.05 was considered to indicate statistical significance.

	Group R/R	Group P/S	Group M/P	*P* value
	*n* = 15	*n* = 13	*n* = 14	
Induction of anesthesia				
Time from medication to loss of consciousness (min)	2.2 [1.7-2.9]	1.5 [1.2-1.8]	2.1 [1.7-2.2]	0.10
Time from medication to less than 60 of Entropy (min)	3.3 [2.6-4.5]	1.7 [1.0-2.8]	3.3 [2.3-3.7]	0.005
Time from medication to tracheal intubation (min)	5.5 [4.4-5.9]	5.0 [4.7-5.8]	5.4 [4.3-6.2]	0.96

End of anesthesia				
Time from discontinuation of medication to extubation (min)	11.7 [7.2-13.3]	9.5 [7.0-11.0]	11.8 [11.3-15.1]	0.03
Time from the end of surgery to extubation (min)	11.9 [6.6-18.7]	16.0 [11.2-20.2]	12.9 [8.8-16.0]	0.38
Time from extubation to discharge from operating room (min)	15.0 [9.1-21.5]	17.7 [15.0-20.0]	19.2 [15.5-20.9]	0.42

Awakening from anesthesia				
Entropy value when flumazenil is administered	30 [26-36]	—	—	
Time from flumazenil administration to Entropy exceeding 90 (min)	2.5 [1.0-3.2]	—	—	

**Table 4:** Changes in mean PA pressure, SvO2, and CI during surgery. There was no difference between the three groups. Values are shown as the median [interquartile range] or number (%). A *P* value < 0.05 was considered to indicate statistical significance. BAV: balloon aortic valvuloplasty; mPA: mean pulmonary artery pressure; SvO2: mixed venous oxygen saturation; CI: cardiac index.

	Group R/R	Group P/S	Group M/P	*P* value
	*n* = 15	*n* = 13	*n* = 14	
mPA				
At the start of the surgery	16.9 [15.9-19.1]	19.9 [17.0-20.7]	19.3 [15.6-22.4]	0.39
15 minutes after surgery	17.9 [16.6-20.3]	19.8 [18.4-21.8]	19.0 [15.4-20.5]	0.48
5 minutes before BAV	18.3 [16.4-20.2]	20.7 [17.8-21.7]	19.5 [17.4-22.3]	0.45
At the end of surgery	17.3 [15.2-20.4]	18.8 [18.6-20.4]	18.6 [15.9-21.2]	0.55
SvO_2_				
At the start of the surgery	71.5 [70.0-79.3]	78.5 [76.3-81.8]	75.0 [71.5-78.5]	0.45
15 minutes after surgery	75.0 [71.8-77.8]	78.0 [76.8-83.0]	77.0 [70.5-79.0]	0.07
5 minutes before BAV	73.5 [67.3-76.5]	78.5 [76.5-81.3]	74.5 [71.8-79.8]	0.34
At the end of surgery	75.0 [71.0-80.0]	82.0 [78.0-84.5]	75.5 [72.8-79.5]	0.05
CI				
At the start of the surgery	2.6 [2.2-2.9]	2.1 [1.9-2.3]	2.0 [1.8-2.5]	0.25
15 minutes after surgery	2.4 [2.2-2.5]	2.5 [2.1-2.9]	2.3 [2.0-2.6]	0.67
5 minutes before BAV	2.1 [2.0-3.0]	2.3 [2.1-2.7]	2.3 [2.2-2.7]	0.64
At the end of surgery	2.1 [1.8-2.3]	2.4 [2.2-2.6]	2.1 [1.7-2.4]	0.27

## Data Availability

The data used to support the findings of this study are available from the corresponding author upon request.

## References

[1] Rogers W. K., McDowell T. S. (2010). Remimazolam, a short-acting GABA(a) receptor agonist for intravenous sedation and/or anesthesia in day-case surgical and non-surgical procedures. *IDrugs*.

[2] Masui K. (2020). Remimazolam besilate, a benzodiazepine, has been approved for general anesthesia!!. *Journal of Anesthesia*.

[3] Wiltshire H. R., Kilpatrick G. J., Tilbrook G. S., Borkett K. M. (2012). A placebo- and midazolam-controlled phase I single ascending-dose study evaluating the safety, pharmacokinetics, and pharmacodynamics of remimazolam (CNS 7056): part II. Population pharmacokinetic and pharmacodynamic modeling and simulation. *Anesthesia and Analgesia*.

[4] Doi M., Morita K., Takeda J., Sakamoto A., Yamakage M., Suzuki T. (2020). Efficacy and safety of remimazolam versus propofol for general anesthesia: a multicenter, single-blind, randomized, parallel-group, phase IIb/III trial. *Journal of Anesthesia*.

[5] Doi M., Hirata N., Suzuki T., Morisaki H., Morimatsu H., Sakamoto A. (2020). Safety and efficacy of remimazolam in induction and maintenance of general anesthesia in high-risk surgical patients (ASA class III): results of a multicenter, randomized, double-blind, parallel-group comparative trial. *Journal of Anesthesia*.

[6] Chen S., Wang J., Xu X. (2020). The efficacy and safety of remimazolam tosylate versus propofol in patients undergoing colonoscopy: a multicentered, randomized, positive-controlled, phase III clinical trial. *American Journal of Translational Research*.

[7] Nishimura R. A., Otto C. M., Bonow R. O. (2017). 2017 AHA/ACC focused update of the 2014 AHA/ACC guideline for the Management of Patients with Valvular Heart Disease: a report of the American College of Cardiology/American Heart Association Task Force on Clinical Practice Guidelines. *Journal of the American College of Cardiology*.

[8] Smith C. R., Leon M. B., Mack M. J. (2011). Transcatheter versus surgical aortic-valve replacement in high-risk patients. *The New England Journal of Medicine*.

[9] Nakanishi T., Sento Y., Kamimura Y., Tsuji T., Kako E., Sobue K. (2021). Remimazolam for induction of anesthesia in elderly patients with severe aortic stenosis: a prospective, observational pilot study. *BMC Anesthesiology*.

[10] Salmasi V., Maheshwari K., Yang D. (2017). Relationship between intraoperative hypotension, defined by either reduction from baseline or absolute thresholds, and acute kidney and myocardial injury after noncardiac surgery: a retrospective cohort analysis. *Anesthesiology*.

[11] Wesselink E. M., Kappen T. H., Torn H. M., Slooter A. J. C., van Klei W. A. (2018). Intraoperative hypotension and the risk of postoperative adverse outcomes: a systematic review. *British Journal of Anaesthesia*.

[12] Claeys M. A., Gepts E., Camu F. (1988). Haemodynamic changes during anaesthesia induced and maintained with propofol. *British Journal of Anaesthesia*.

[13] Franco A., Gerli C., Ruggeri L., Monaco F. (2012). Anaesthetic management of transcatheter aortic valve implantation. *Annals of Cardiac Anaesthesia*.

[14] Christ M., Sharkova Y., Geldner G., Maisch B. (2005). Preoperative and perioperative care for patients with suspected or established aortic stenosis facing noncardiac surgery. *Chest*.

[15] Hiltebrand L. B., Koepfli E., Kimberger O., Sigurdsson G. H., Brandt S. (2011). Hypotension during fluid-restricted abdominal surgery: effects of norepinephrine treatment on regional and microcirculatory blood flow in the intestinal tract. *Anesthesiology*.

[16] Yang H. S., Song B. G., Kim J. Y., Kim S. N., Kim T. Y. (2013). Impact of propofol anesthesia induction on cardiac function in low-risk patients as measured by intraoperative Doppler tissue imaging. *Journal of the American Society of Echocardiography*.

[17] Han D. W., Chun D. H., Kweon T. D., Shin Y. S. (2008). Significance of the injection timing of ephedrine to reduce the onset time of rocuronium. *Anaesthesia*.

[18] Ezri T., Szmuk P., Warters R. D., Gebhard R. E., Pivalizza E. G., Katz J. (2003). Changes in onset time of rocuronium in patients pretreated with ephedrine and esmolol--the role of cardiac output. *Acta Anaesthesiologica Scandinavica*.

[19] Dingemanse J., van Gerven J. M., Schoemaker R. C. (1997). Integrated pharmacokinetics and pharmacodynamics of Ro 48-6791, a new benzodiazepine, in comparison with midazolam during first administration to healthy male subjects. *British Journal of Clinical Pharmacology*.

[20] Kilpatrick G. J., McIntyre M. S., Cox R. F. (2007). CNS 7056. *Anesthesiology*.

[21] Ibrahim A. E., Taraday J. K., Kharasch E. D. (2001). Bispectral index monitoring during sedation with sevoflurane, midazolam, and propofol. *Anesthesiology*.

[22] Glass P. S., Bloom M., Kearse L., Rosow C., Sebel P., Manberg P. (1997). Bispectral analysis measures sedation and memory effects of propofol, midazolam, isoflurane, and alfentanil in healthy volunteers. *Anesthesiology*.

[23] Schnider T. W., Minto C. F., Shafer S. L. (1999). The influence of age on propofol pharmacodynamics. *Anesthesiology*.

[24] Kurita T., Morita K., Kazama T., Sato S. (2002). Influence of cardiac output on plasma propofol concentrations during constant infusion in swine. *Anesthesiology*.

[25] Upton R. N., Martinez A. M., Grant C. (2009). Comparison of the sedative properties of CNS 7056, midazolam, and propofol in sheep. *British Journal of Anaesthesia*.

[26] Upton R. N., Somogyi A. A., Martinez A. M., Colvill J., Grant C. (2010). Pharmacokinetics and pharmacodynamics of the short-acting sedative CNS 7056 in sheep. *British Journal of Anaesthesia*.

[27] Stiegler M. P., Tung A. (2014). Cognitive processes in anesthesiology decision making. *Anesthesiology*.

